# Maternal Mortality and Morbidity by Cause in Provinces of Iran, 1990 to 2019: An Analysis for the Global Burden of Disease Study 2019

**DOI:** 10.34172/aim.2022.93

**Published:** 2022-09-01

**Authors:** Sadaf G. Sepanlou, Hossein Rezaei Aliabadi, Reza Malekzadeh, Mohsen Naghavi, GBD 2019 Iran Maternal Collaborators

**Affiliations:** ^1^Digestive Disease Research Institute, Tehran University of Medical Sciences, Tehran, Iran; ^2^Bam University of Medical Sciences, Bam, Iran; ^3^Institute for Health Metrics and Evaluation, School of Medicine, University of Washington, Seattle, USA

**Keywords:** Global Burden of Disease, Iran, Maternal health services, Maternal mortality, Sustainable development

## Abstract

**Background:**

Since 1990, maternal mortality ratio (MMR) has significantly decreased in Iran. However, estimates for mortality and morbidity by cause at subnational scale are not available.

**Methods:**

This study is part of the Global Burden of Diseases study (GBD) 2019. Here we report maternal mortality and morbidity by age and cause across 31 provinces of Iran from 1990 to 2019.

**Results:**

Since 1990, MMR declined from 44.5 (95% UI: 38.6-50.1) to 15.9 (14.7–17.3) per 100000 live births in Iran. In 1990 MMR ranged from 18.5 (11.2–26.4) to 76.9 (38.4–114.7) per 100000 live births across provinces. Respective figures for 2019 were 7.1 (5.2–9.3) to 34.0 (25.1–44.7) per 100000 live births. In 2019, MMR was higher in young women (aged 10 to 14) and older women (aged 45 or more). Percentages of deaths under 25 years was 24.8% in 1990 and 16.0% in 2019. There was remarkable decline in years lost due to premature death (YLL) rates from 1990 to 2019. While the decline was modest for years lived with disability (YLD) rates. Indirect maternal deaths and other maternal deaths ranked first or second in almost all provinces. Ultimately, there was an evident decrease in MMR along with increase in socio-demographic Index (SDI) from 1990 to 2019 in all provinces and an evident convergence across provinces.

**Conclusion:**

MMR has declined to levels much lower than Sustainable Development Goals in all provinces. Although there was a convergence in trends, there are still disparities across provinces. The decline in disabilities caused by maternal disorders is not as significant as mortality, which needs further actions.

## Introduction

 Maternal mortality and disorders are key indicators of development in nations, and are a function of various public health, social, and economic determinants. The important point worth noting is that maternal mortality is avoidable and appropriate policies can substantially reduce the burden of maternal disorders.^1^ During the past four decades the global health community has focused on reducing maternal mortality and morbidity, shown by its choice as one of the eight Millennium Development Goals (MDGs)^2^ and recently chosen as one of the main targets of Sustainable Development Goals (SDGs). The target is to reduce the global maternal mortality ratio (MMR) to less than 70 per 100 000 live births by 2030 in all countries.^3^

 Since 1980, substantial progress has been made in health infrastructure in Iran. With the establishment of primary healthcare system in Iran staffed with trained community health workers (*Behvarz*) mainly in rural areas,^4-6^ the family physician program,^7^ and the health sector evolution plan,^8^ the health care provision is now substantially extensive and much more equitable.^6,9^ Specifically, the National Maternal Mortality Surveillance System was designed in 2000 and established in 2001 throughout Iran.^10^ The results were a remarkable decline in maternal mortality in Iran in the past decades. The World Health Organization estimated a very low MMR of 16 (80% UI: 13-20) per 100 000 live births for Iran in 2017, showing a 67% decrease from an MMR of 48 per 100 000 live births in 2000.^1^ During the same time period, the number of maternal deaths has decreased steadily in Iran from 560 to 250 deaths in 2017.^1^

 Alternatively, similar updated estimates for maternal mortality and morbidity have been made for Iran by the Global Burden of Diseases, Injuries, and Risk Factors (GBD) study. GBD 2019 is a comprehensive and systematic effort that estimated levels and trends of burden caused by 369 diseases and injuries across 204 countries and territories from 1990 to 2019.^11^ Estimates have also been made at subnational level in selected countries including Iran. Despite existing detailed data on national levels and trends of maternal mortality in Iran, sub-national estimates were not assessed and not publicly published before GBD 2019. Additionally, to the best of our knowledge, the non-fatal burden of maternal disorders hasn’t been estimated for Iran so far. Therefore, in the current study, we report the estimates of the GBD at national and sub-national scales for maternal mortality and morbidity in Iran from 1990 to 2019.^11,12^

## Materials and Methods

 This study was part of GBD 2019, which was a systematic effort to estimate the levels, trends, and causes of mortality and morbidity by sex, age, year (1990 to 2019), and location. In this article we report estimates for fatal and non-fatal maternal disorders at national and subnational levels in Iran.^11-13^

 To estimate cause-specific maternal mortality and morbidity, we used data from Iran Vital Registration (VR), Iran Death Registration System, Iran Maternal Mortality Surveillance, Iran censuses, Iran Maternal Mortality Report (2012–2013), results of Reproductive Age Mortality Study (RAMOS) in Iran (1996),^14^ Iran National Integrated Micronutrient Survey (2012), Iran National Health Accounts, Iran Multiple Indicator Cluster Survey, and the published scientific literature on maternal mortality and disorders in Iran. Our systematic literature review for maternal disorders is updated annually and encompasses all aspects of maternal disorder burden estimation.^11,12^

###  Maternal Mortality

 There has been much debate about the definition of maternal deaths. To be classified as maternal, pregnancy needs to be a causal factor in death. It can either have a direct effect (complications of the pregnancy or childbirth, or postpartum complications) or indirect effect (exacerbation of a pre-existing condition). Therefore, accidental or incidental deaths in which pregnancy had no causal role are not classified as maternal deaths.^15^ We included direct and indirect deaths during pregnancy and within 6 weeks of delivery, plus late maternal deaths after 6 weeks up to 1 year after delivery and the fraction of HIV-related deaths aggravated by pregnancy.^15^ We disaggregated maternal deaths into ten causes: 1) maternal hemorrhage, 2) maternal sepsis and other pregnancy-related infections, 3) hypertensive disorders of pregnancy, 4) obstructed labor, 5) abortion, 6) other direct maternal disorders, 7) indirect maternal disorders, 8) ectopic pregnancy, 9) HIV, and 10) late maternal deaths.^11^

 For overall maternal mortality and cause-specific mortality, all data were reviewed in cause of death ensemble models (CODEm). The details are previously published.^16^ Covariates included in the model for overall maternal mortality, their level, and directionality are show in Supplementary file 1 (Table S1 and S2). Outliers were identified as those data where age patterns or temporal patterns were inconsistent with neighboring age groups or locations or where sparse data were predicting implausible overall temporal or age patterns for a given location. All cause-specific maternal mortality data were extracted as MMR (cause-specific deaths per live births). All cause of death (COD) data, along with any sources that reported cause-specific maternal deaths in cause fraction or population rate terms, were converted to MMR using all-cause mortality, population, and age-specific fertility results estimated in GBD 2019.^11,12^ We used spatiotemporal Gaussian process regression (ST-GPR) to estimate MMRs for each of the maternal sub-causes.^11,12^

 Cause-specific estimates were derived by scaling the results from the ST-GPR subcause-specific models scaled in relation to each other to equal one and then multiplying them by the total maternal deaths, corrected for late maternal deaths, for that age group, location, and year. A single parameter proportion model was run in Dismod-MR 2.1, which is a Bayesian meta-regression tool developed for the GBD, for late maternal deaths using the data described above. The final result includes cause fraction and number of maternal deaths due to each cause, by country and province, age group, and year. All cause-specific MMR and proportion data were uploaded to the non-fatal database.^11,12^

###  Maternal Morbidity

 Maternal disorders nonfatal estimation includes disability due to seven of ten maternal mortality sub-causes, excluding indirect maternal deaths, late maternal deaths, and maternal deaths aggravated by HIV/AIDS, which did not have any estimated disability.^11,12^

 All data were either extracted as incidence ratio (number of events / live birth) or, if data were only available with population as the denominator, they were converted to incidence ratio using GBD 2019 age-specific fertility rate (ASFR; number of live births / population). The reason is that most literature and surveillance data are expressed in terms of number of events per live birth rather than per population. Hospital and claims data, which were centrally processed for all GBD 2019 causes to have population as the denominator, were transformed to have livebirths as the denominator by dividing by ASFR (live births per population).

 The first step of data processing was age-sex splitting. For any datum that did not entirely fit within a GBD age group or was for both sexes combined, the observation was split to be multiple age-specific and sex-specific data points based on the age and sex pattern predicted by GBD 2017 DisMod-MR 2.1 models. It is our intention to update this age-sex splitting with each cycle of GBD. The second step was cross-walking all data from alternate to reference definitions. We adjusted data to the reference category for each cause by age using Meta-Regression - Bayesian, Regularized, Trimmed (MR-BRT), a meta-analytic tool developed for GBD 2019. The details of each of the crosswalks are previously published.^11,12^ All data sources that only reported event rates for severe maternal morbidity or “near miss” were excluded as a reliable crosswalk model could not be developed.

 We estimated the incidence ratio of each category of pregnancy complications for each age-sex-location-year in the GBD 2019 location hierarchy using DisMod-MR 2.1. After completion of DisMod-MR 2.1 models, all age-specific ratios were then converted to incidence rates by multiplying by ASFR and then to prevalence rates by applying a global assumed duration of disability for each type of pregnancy complications.^11,12^ We quantified disability weights for each maternal disorder and finally calculated years lived with disability (YLD) for each 7 maternal disorders. Disability-adjusted life years (DALYs) were the sum of YLDs and years lost due to premature death (YLLs) previously estimated for maternal mortality.^11,12^ Ultimately, we report DALYs in terms of both numbers and rates. We report 95% uncertainty intervals (UIs) for all estimates. All-cause and cause-specific mortality and morbidity estimation components are based on 1000 draws, or simulations, by age, sex, location, and year. Point estimates were derived from the mean of the draws, and 95% UIs were calculated as the 2.5^th^ and 97.5^th^ percentiles of the draws.

###  Socio-demographic Index

 We used the socio-demographic index (SDI) to determine the relationship between the development level of a province and MMR. In GBD 2017, the SDI was revised to better reflect the development status of countries and provinces. The SDI ranges from 0 (worst) to 1 (best) and is a composite measure of the total fertility rate in women under the age of 25 years, mean education for individuals aged 15 years and older, and lag-distributed income per capita.^17-19^ We report 95% UIs for all estimates.

## Results

###  National Estimates for Maternal Mortality

**Figure 1:**
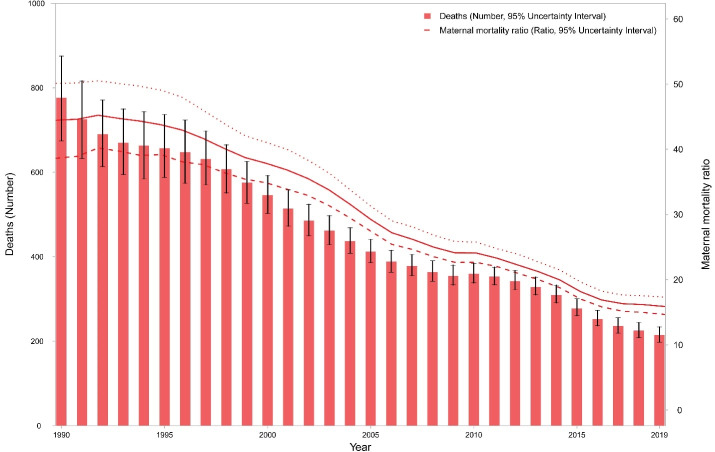
Number of Maternal Deaths and Maternal Mortality Ratio from 1990 to 2019 in Iran

 In 1990, 776 (95% UI: 674–875) deaths occurred due maternal disorders in Iran, which decreased to 214 (198–234) deaths in 2019. During this time period, MMR dropped from 44.5 (38.6–50.1) to 15.9 (14.7–17.3) per 100 000 live births (Table 1 and Figure 1). Meanwhile, the number of DALYs were 55,571 (48,579–63,072) years in 1990 and 17,789 (15,309–20.766) years in 2019. Respective figures for age-standardized DALY rates were 210.4 (184.6–237.3) and 36.9 (31.7–43.3) per 100 000 females (Figure S1).

**Table 1:** Maternal Mortality Ratio per 100 000 Live Births Across Provinces of Iran and the Percent Change from 1990 to 2019

**Iran & Provinces**	**MMR 1990 (95% UI)**	**MMR 2019 (95% UI)**	**Percent Change**
Iran	44.5 (38.6, 50.1)	15.9 (14.7, 17.3)	-64.3 (-69.1, -57.4)
Alborz	27.4 (8.6, 41.8)	11 (8.1, 15)	-59.8 (-76.4, 28.5)
Ardebil	44.7 (23.6, 64.5)	14.2 (10.7, 18.1)	-68.3 (-79.2, -35.9)
Bushehr	65 (31.7, 92.8)	17.5 (13.5, 21.8)	-73.1 (-82.8, -44.5)
Chahar Mahaal and Bakhtiari	23.9 (14.7, 34.9)	7.1 (5.2, 9.3)	-70.1 (-82.3, -48.2)
East Azarbayejan	49.8 (32.4, 72.7)	14.1 (10.3, 19.3)	-71.7 (-83.4, -52.2)
Fars	48.6 (24.2, 72.7)	18.6 (13.4, 25.3)	-61.6 (-77.6, -19.7)
Gilan	34.4 (17.6, 51.5)	11.6 (8.3, 15.6)	-66.2 (-79.2, -29.2)
Golestan	35.9 (22.7, 51.7)	15.2 (11.5, 20)	-57.8 (-73.5, -25.7)
Hamadan	43.8 (29.8, 61.7)	15.5 (11.4, 20.7)	-64.6 (-77.3, -42)
Hormozgan	66.7 (17.1, 102.4)	20.5 (15.3, 26.7)	-69.2 (-81.2, 10.1)
Ilam	18.5 (11.2, 26.4)	9.5 (7.4, 12.2)	-48.3 (-67.3, -8.2)
Isfahan	41.8 (14.6, 62.3)	17.2 (12.6, 23.1)	-58.9 (-75.5, 33.9)
Kerman	59.3 (36.2, 87.7)	20 (14.9, 26.2)	-66.3 (-79.2, -41.2)
Kermanshah	57.8 (36.8, 84.8)	20.4 (15, 27.3)	-64.7 (-78.5, -34.2)
Khorasan-e-Razavi	62 (26.8, 91.3)	18 (13.6, 23.3)	-71.1 (-81.5, -34.2)
Khuzestan	36.3 (22.8, 52.8)	15.9 (11.8, 21.3)	-56.4 (-72.6, -26)
Kohgiluyeh and Boyer-Ahmad	43.6 (27, 61.9)	20.4 (14.4, 28.2)	-53.1 (-72.3, -14.4)
Kurdistan	60.6 (39.5, 87.9)	16.6 (12.5, 21.9)	-72.5 (-82.7, -54.4)
Lorestan	32.4 (20.7, 48.4)	13.6 (10, 18.3)	-57.9 (-74.6, -26.7)
Markazi	35.9 (22.9, 52.6)	10.9 (8.2, 14.8)	-69.5 (-81.6, -43.6)
Mazandaran	24.3 (15.6, 36.9)	11.1 (8.1, 14.8)	-54.5 (-72.9, -21.7)
North Khorasan	44.4 (30.7, 64.7)	13.9 (10.5, 17.8)	-68.8 (-79.8, -50.6)
Qazvin	54.7 (32.3, 78.6)	18 (13.3, 23.1)	-67.2 (-79.9, -38.5)
Qom	36.4 (8.5, 53.7)	9.1 (6.8, 12.2)	-74.8 (-84.6, 7.3)
Semnan	76.9 (38.4, 114.7)	23.3 (17.2, 30.5)	-69.7 (-81.5, -35.7)
Sistan and Baluchistan	70.5 (17.1, 110.7)	34 (25.1, 44.7)	-51.7 (-72.2, 89.5)
South Khorasan	42.8 (30.8, 58.2)	15.4 (11.6, 20.1)	-64 (-75.9, -45.4)
Tehran	30.4 (19.9, 44.8)	8.5 (6, 11.8)	-72.2 (-83.6, -51.6)
West Azarbayejan	55.7 (38.6, 78.6)	14.7 (11.1, 19.4)	-73.7 (-83.3, -59.6)
Yazd	45 (29.8, 65.3)	17.6 (12.5, 23.5)	-60.8 (-74.9, -34.5)
Zanjan	27.5 (17.2, 39.3)	8 (6.1, 10.6)	-71 (-81.3, -49.8)

MMR, maternal mortality ratio.

###  Provincial Estimates for Maternal Mortality

**Figure 2:**
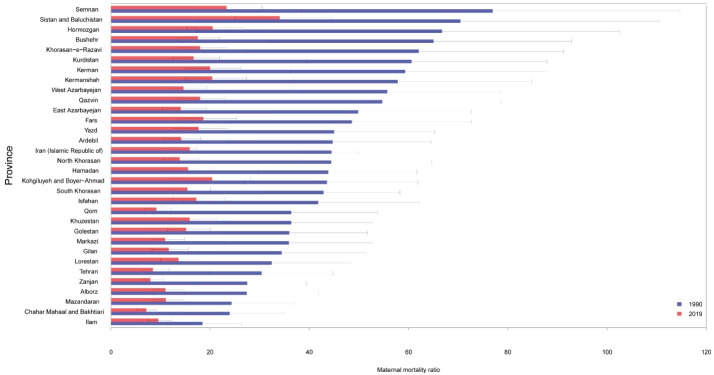
Maternal Mortality Ratio Across Provinces of Iran in 1990 and 2019

**Figure 3:**
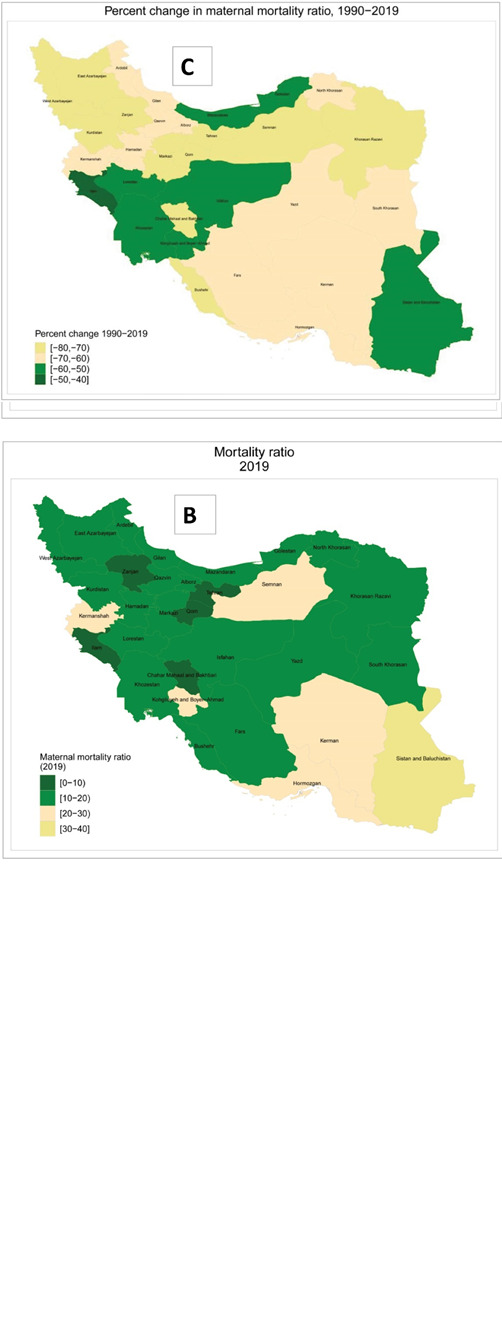
Maternal Mortality Ratio in province of Iran. (A) Maternal Mortality Ratio across provinces of Iran in 1990. (B) Maternal Mortality Ratio across provinces of Iran in 2019. (C) Percent change in Maternal Mortality Ratio across provinces of Iran from 1990 to 2019

 In 1990, MMR ranged from 18.5 (11.2–26.4) per 100 000 live births in Ilam to 76.9 (38.4–114.7) per 100 000 live births in Semnan. Respective figures for 2019 were 7.1 (5.2–9.3) in Chahar Mahal and Bakhtiari to 34.0 (25.1–44.7) in Sistan and Balouchistan (Figure 2, Figure 3A and 3B, and Table 1). The highest percent decrease in MMR from 1990 to 2019 was observed in Qom [-74.8% (-84.6–7.3)] and the lowest percent decrease occurred in Ilam [-48.3% (-67.3, -8.2) (Table 1 and Figure 3C). Figures S2 and S3 demonstrate the age-standardized rates of DALYs across provinces in 1990 and 2019.

**Figure 4:**
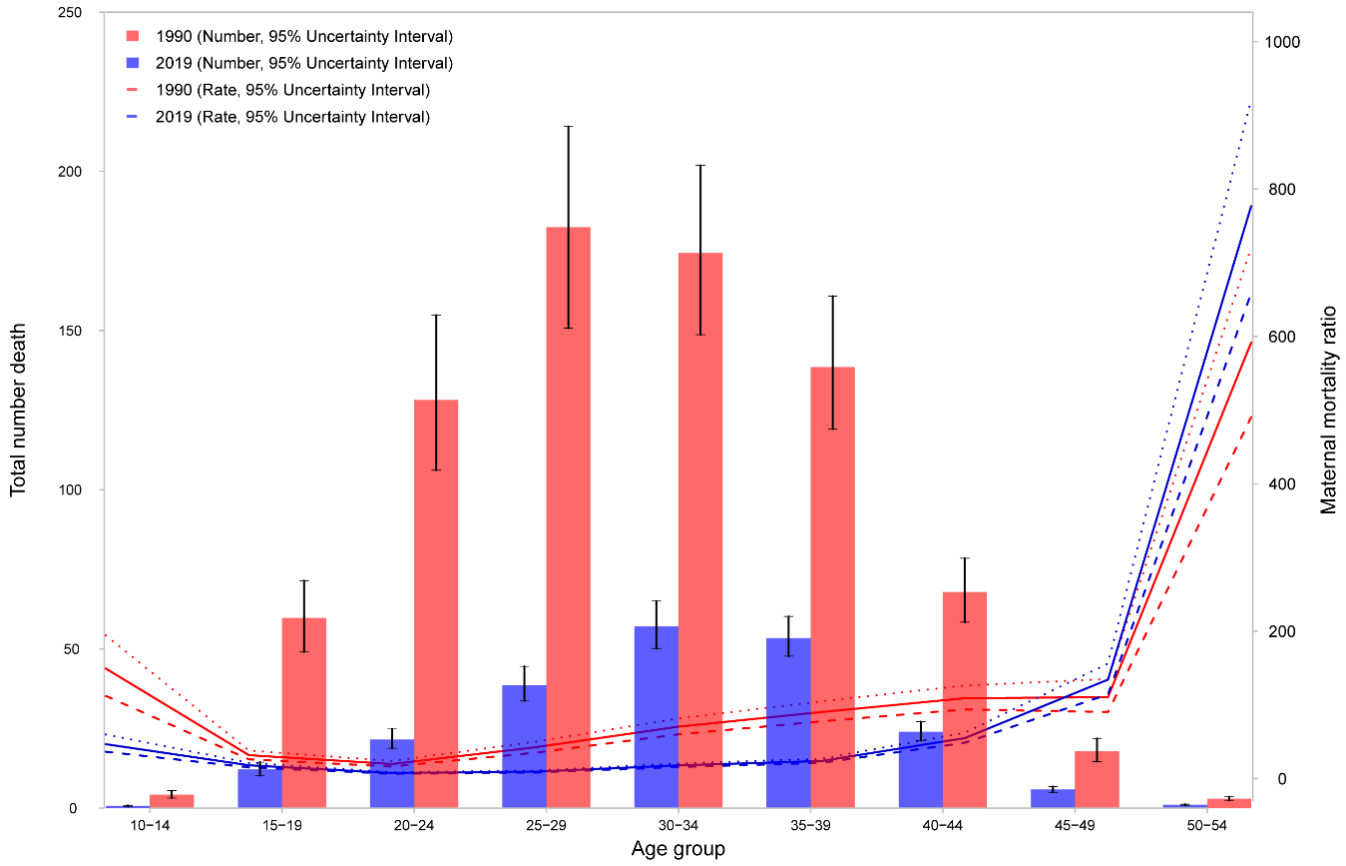
The age pattern of Maternal Mortality Ratio and Maternal Deaths in Iran in 2019

###  Age Pattern of Maternal Mortality

 The age pattern of maternal mortality shows high numbers in middle aged women (30 to 39 years) in 2019. However, Figure 4 shows that MMRs are high in younger women (aged 10 to 14 years) and older women (aged 45 and more). The pattern was similar in 1990 though the highest.

**Figure 5:**
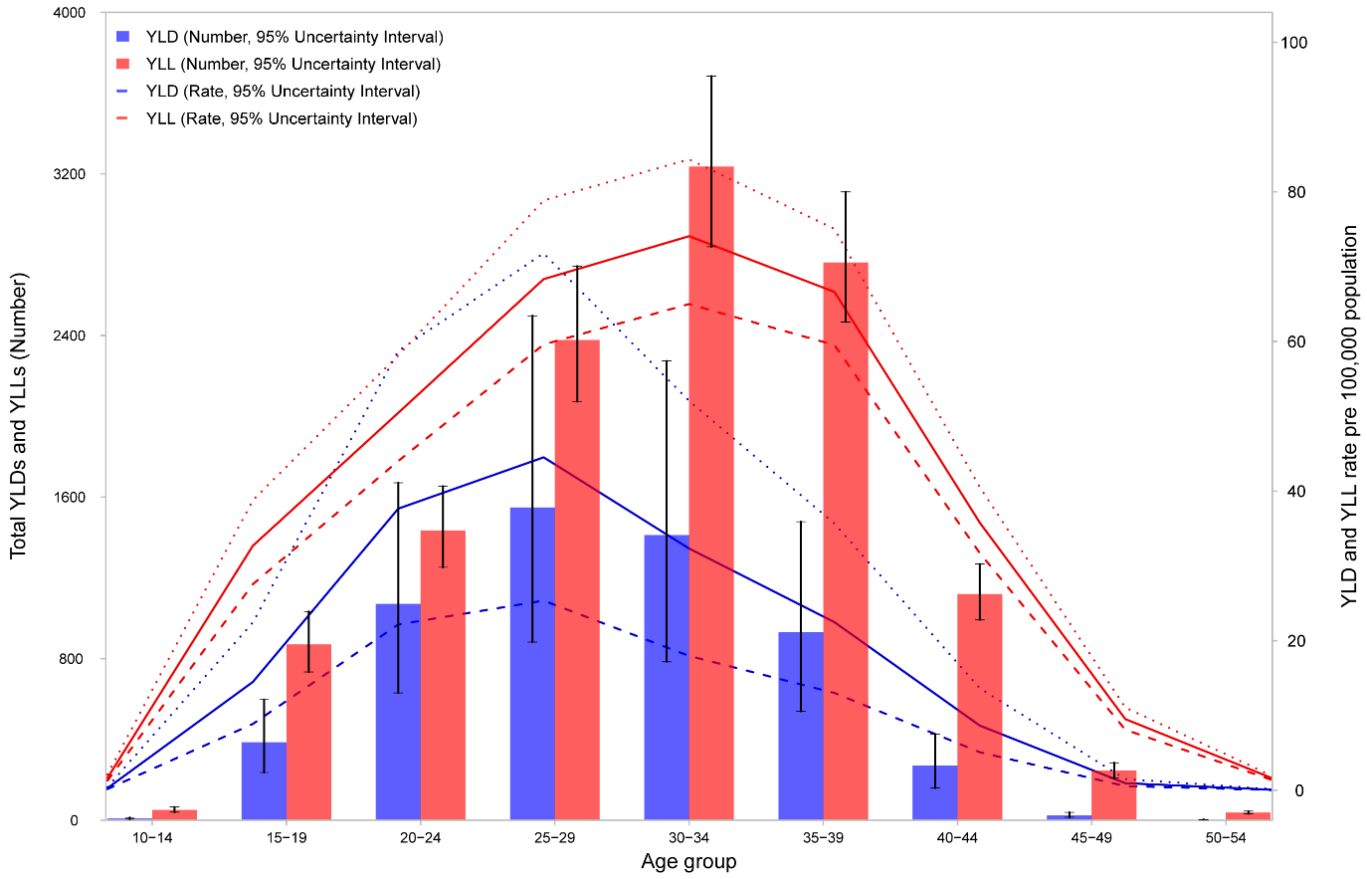
The Age Pattern of YLL and YLD Number and Rates due to Maternal Disorders in 2019

 number of deaths belonged to age group of 25 to 29 years in 1990 and the age group of 35 to 39 years in 2019. However, the MMR among the age group of 10 to 14 years in 1990 was higher than 2019, while the MMR among the age group of 50 to 54 years in 2019 was higher than 1990. Percentages of deaths under 25 years was 24.8% in 1990 and 16.0% in 2019. The age pattern of DALY numbers and rates in 2019 are demonstrated in Figure S4. DALY counts were highest in the age group of 30 to 34 years and DALY rates were highest in the age group of 25 to 29 years.

###  Maternal Morbidity

Figure 5 shows the age pattern of YLLs and YLDs for maternal disorders. In all age groups, YLLs were higher than YLDs. YLLs number and rates peaked in mothers aged 30 to 34 years while YLDs peaked in the age group of 25 to 29 years. Age-standardized YLL rate decreased from 174.8 (152.4–196.8) per 100 000 in 1990 to 24.8 (22.9–27.1) per 100 000 in 2019. However, the change in age-standardized YLD rates was modest, from 35.6 (22.0–51.5) per 100 000 in 1990 to 12.1 (7.4–18.1) per 100 000 in 2019. Figures S5 and S6 show the trend in number and age-standardized rates per 100 000 of YLLs and YLDs due to maternal disorders from 1990 to 2019. Figure S7 demonstrates the share of YLLs and YLDs out of DALYs for maternal disorders by cause in 1990 and 2019. For most causes, the share of YLL has decreased from 1990. The two exceptions are maternal obstructed labor and uterine rupture, and maternal hemorrhage.

**Figure 6:**
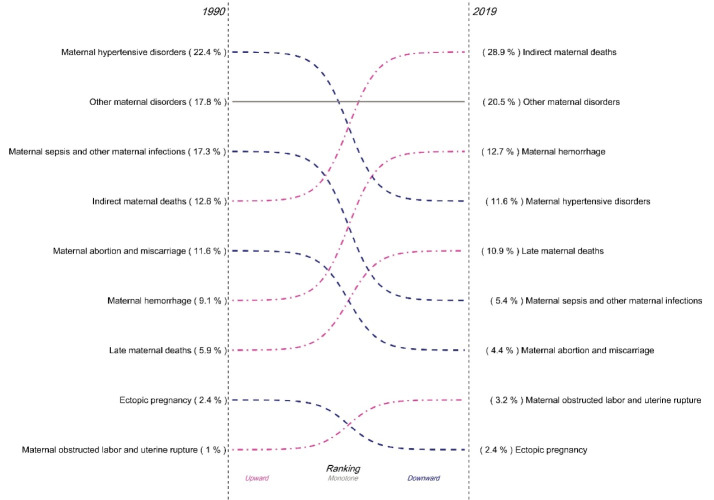
The Rank of Maternal Mortality Causes in 1990 and 2019

###  Maternal Mortality by Cause at National Level

Figure 6 demonstrates the share of each cause of death out of the total deaths caused by maternal disorders in Iran in 1990 and 2019. During the time period, the share of indirect maternal deaths out of the total maternal deaths more than doubled from 12.6% to 28.9%, the share of other maternal disorders increased from 17.8% to 20.5%, the share of maternal hemorrhage increased slightly from 9.1% to 12.7%, and the share of late maternal deaths increased from 5.9% to 10.9%. On the contrary, the share of maternal hypertensive disorders almost halved from 22.4% to 11.6%, the share of maternal sepsis and other maternal infections decreased by more than 68% from 17.3% to 5.4%, and the share of maternal abortion and miscarriage declined from 11.6% to 4.4%. The share of deaths due to maternal obstructed labor and uterine rupture in 1990 was just one percent, which increased to 3.1% in 2019. The share of mortality due to ectopic pregnancy didn’t change (2.4% in both years).

**Figure 7:**
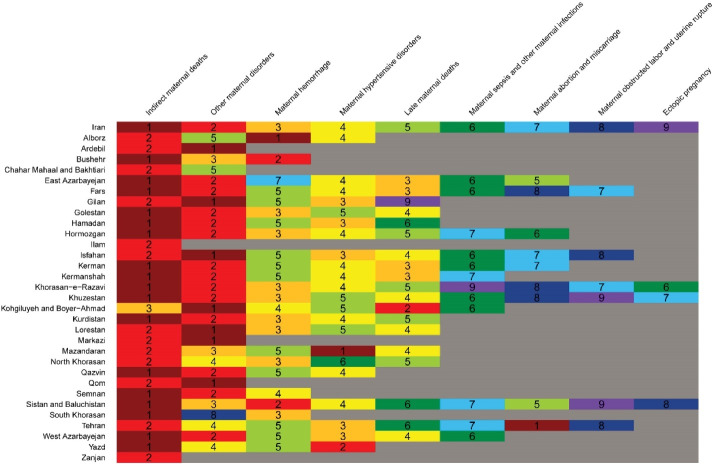
The Heatmap Showing the Ranks of Maternal Mortality by Cause Across Provinces of Iran in 2019. The gray shaded areas show no deaths. Each color in the first row shows the rank of each maternal cause at national level, which is compared to their ranks in provincial level in the following rows.

###  Maternal Mortality by Cause at Provincial Level

Figure 7 demonstrates the order of maternal mortality causes in Iran and across provinces in 2019. The first five causes are almost the same across provinces. Indirect maternal deaths ranked first in 18 provinces and second in 7 provinces. Other maternal deaths also ranked either first or second in 27 provinces. Maternal hemorrhage, maternal hypertensive disorders, and late maternal deaths ranked 3^rd^ to 5^th^ in almost all provinces. Maternal deaths aggravated by HIV/AIDS were actually zero in all provinces.

**Figure 8:**
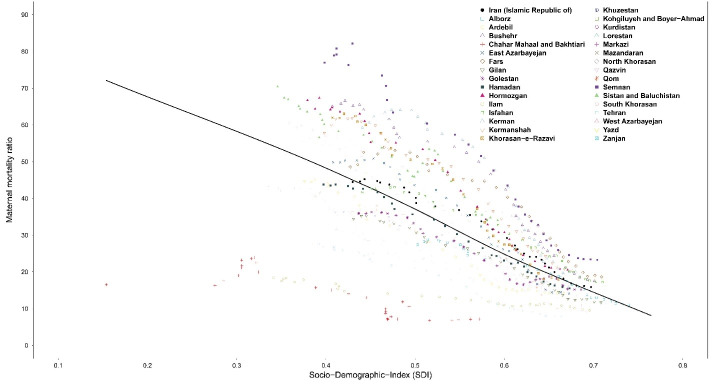
The Trend in Maternal Mortality Ratio across Provinces along with Increase in SDI from 1990 to 2019

###  Sociodemographic Index

 Ultimately, we explored the association of SDI with MMR from 1990 to 2019 (Figure 8). There was an evident decrease in MMR along with increase in SDI from 1990 to 2019 in all provinces. There was also an evident convergence in MMRs across provinces implying decreasing inequality in distribution of maternal mortality since 1990. The same trend was observed for age-standardized DALY rates due to maternal disorders from 1990 to 2019 (Figure S8).

## Discussion

 The results of GBD 2019 demonstrated a substantial decline in MMR, the number of maternal deaths, and the DALYs due to maternal disorders in Iran since 1990. Rates decline along with increase in SDI and the decreasing trends have converged during this time period. Results show that all provinces in Iran have already achieved the MMR below 70 per 100 000 live births set in targets of SDG.^20^ Our results for national level of MMR are quite compatible with those released by WHO in 2019 and the results of GBD 2013.^1,15^

 At the regional level, the MMR in Iran (15.9 per 100 000 live births) is much lower than the ratio across the entire region of north Africa and Middle East (with an MMR of 94.3 per 100 000 live births). Among all countries in north Africa and Middle East, Iran was the second country with lowest MMR in 2019, outpaced only by Kuwait, with an MMR of 5.1 per 100 000 live births. Even at the global level, the MMR estimate in Iran was much lower than global estimates (145.2 per 100 000 live births in 2019) and surprisingly much lower than a high-income country such as the United States of America (25.9 per 100 000 live births).

 The age pattern of maternal mortality demonstrated that MMRs are higher in youngest and oldest age groups, which is an already known observation. However, another observation is that MMR has declined in youngest age groups since 1990 while we can observe an increase in MMR in the oldest age group during the past three decades. Together with decreased percentage of deaths among women less than 25 years old, we can conclude that child marriages have declined in Iran, while pregnancy among older age groups has increased.

 The drivers of improvements in underlying causes of maternal deaths have important clinical, public health, and policy implications. There are various health and non-health determinants for maternal mortality and morbidity such as women’s literacy,^21^ urbanization, rural access roads, access to emergency and maternal health care especially in deprived areas, health costs, household wealth,^22^ health insurance coverage, and other health and non-health infrastructure that affect timeliness and quality of maternal care and the overall degree of development in nations.^15,23-25^ The absolute numbers of deaths due to all causes of maternal death have decreased in all provinces of Iran, with maternal hemorrhage, maternal hypertensive disorders, maternal sepsis and other maternal infections, and maternal abortion and miscarriage constituting over 60% of all deaths in 1990 and responsible for just slightly over 34% of all deaths in 2019. The reduction in direct causes of these deaths can be attributed to the integration of the family physician program and the increase in density of physicians and midwives in rural deprived areas of Iran.^26-28^ Another determinant can be reduced malnutrition and anaemia during pregnancy.^29^ Other drivers can be provision of calcium and micronutrient supplementation, discouraging early motherhood, encouraging skilled birth attendance and in-facility delivery, and reducing unsafe abortion, which constitute the care that is provided by the community health care workers, “Behvarz’s” and the family physicians.^30-32^ However, the most important driver of improvement in maternal health in Iran can be attributed to implementation of the National Maternal Mortality Surveillance System throughout Iran in 2001.^10^ In this surveillance system, all cases of maternal deaths are notified and the maternal mortality committee explores the cause of death and the probable defect in health care provision that may have led to mortality due to pregnancy in order to prevent similar cases. With the implementation of this system, many common and repetitious causes of maternal deaths were prevented.

 The Iranian health care system has observed one of the most successful achievements in improving maternal health at the global level and even in comparison with high-income countries such as the United States of America and countries in Western Europe. These achievements have numerous elements and drivers, mainly the effective primary health care system, the universal health coverage, and other aforementioned central and unified policies that are made and implemented at the level of the Ministry of Health. The system, however, now faces new challenges due to increased share of other direct, indirect, and late maternal causes of death, which is consistent with the global and national epidemiological transition,^33,34^ and suggests that the health system in Iran may not appropriately prepared to meet the needs of an increasing number of pregnant women with high-risk pregnancies and pre-existing conditions. Many risks and diseases such as obesity,^35,36^ diabetes,^37^ hypertension,^38^ chronic kidney disease,^39,40^ and their clustering increase the risk of mortality during pregnancy.^41^ These indirect causes of maternal death are gaining importance.^42^ Health system policies in Iran should be made capable of confronting these challenges through facilitating the recruitment and training of the perioperative skilled staff,^43^ and further investment in providing adequate infrastructural resources, and service packages integrated in the national surveillance system to identify and follow women who are at great risk of life-threatening puerperal and postpartum complications. Surveillance of late maternal mortality ( > 42 days but < 1 year) should be included in surveys and censuses in near future.

 The above mentioned policies have largely reduced the risk of life-threatening complications of pregnancy in Iran, but the complications will not be eliminated altogether. The results of our study show that despite significant decrease in maternal mortality, there was almost no improvement in disability caused by complications of pregnancy. Assuming that maternal mortality will continue to decrease, severe maternal morbidity or so-called near miss cases are likely to increase, some of which might be expected to lead to increased late maternal death.^44-46^ Maternal near miss is defined as “a woman who nearly died but survived a complication that occurred during pregnancy, childbirth or within 42 days of termination of pregnancy”.^44,45^ Tools have been developed in the format of WHO maternal near miss approach for assessing the management of severe maternal morbidity,^47^ and moving beyond essential interventions is a necessity to improve the quality of care.^48^ Studies demonstrate that the quality of care for severe maternal morbidity using the WHO maternal near miss tool is not satisfactory in Iran.^49-51^

 Despite the shortcomings, the overall successful performance of the health system in Iran is reflected in the much lower observed versus expected MMR based on SDI among the neighboring countries in north Africa and Middle East. The policies implemented were tailored to the specific cultures in Iran, which are similar to other countries in the region and can be adopted in the regional scale.

 To the best of our knowledge, the current study is the first that addresses maternal mortality and morbidity by cause and by province in Iran during the past three decades. However, our study has certain limitations as well. The main limitation of GBD studies is lack of adequate and reliable data by time and location. Second, there is still no definitive solution for estimating the interaction of HIV and pregnancy in death and we have very probably underestimated the effect of HIV on maternal mortality. HIV has been described by some sources as a risk factor for late maternal death. If this description is true, these deaths might not be captured appropriately, because neither reproductive health surveys nor demographic and health surveys quantify late maternal death. Third, due to lack of data it was not possible to estimate the contribution of infections other than HIV. Finally, we have estimated UIs for each component of the analysis. CODEm provides confirmation that the UIs for the maternal mortality model have a data coverage of 97·9%, so they could be slightly overestimated.

 Future research should be focused on exploring the determinants of maternal disorders such as malnutrition, and the medical and non-medical cost of care for maternal disorders at national and subnational level in Iran. Future policies should be focused on enhancing the quality and quantity of existing data on levels and trends of the burden due to maternal disorders at subnational burden. Future policies should additionally aim at ensuring timely access to antenatal care, skilled birth attendance, postnatal care, emergency obstetric care, and reproductive health care.

 In conclusion, there has been a considerable decline in maternal mortality in all provinces of Iran during the past three decades. Inequality between provinces has substantially decreased. However, there has not been any significant improvement in maternal disability. The health system of Iran currently requires an impetus to increase the quality of care for reducing the burden due to indirect and late maternal causes of mortality and morbidity. With the continuation of COVID-19 pandemic and sanctions on Iran, the health system of Iran may be overwhelmed, which may negatively influence maternal health specifically among vulnerable populations.

## Supplementary files


Supplementary file 1 contains Tables S1-S2 and Figures S1-S8.
