# Supplementary material for: Maternal Mortality and Morbidity by Cause in Provinces of Iran, 1990 to 2019: An Analysis for the Global Burden of Disease Study 2019
Source: Arch Iran Med. 2022 Sep 1;25(9):578–90. doi: 10.34172/aim.2022.93 (PMC10685769; doi:10.34172/aim.2022.93)
Supplement: Supplementary file 1 — contains Tables S1-S2 and Figures S1-S8. [file aim-25-578-s001.pdf]

Arch Iran Med. September 2022;25(9):578-590
10.34172/aim.2022.93

Maternal Mortality and Morbidity by Cause in Provinces of Iran, 1990 to 2019: An Analysis for the Global Burden of Disease Study 2019

Sadaf G. Sepanlou, MD, MPH, PhD[1]; Hossein Rezaei Aliabadi, MSc[2]; Reza Malekzadeh, MD[1*]; Mohsen Naghavi, MD[3*]; GBD 2019 Iran Maternal Collaborators[#]

[1]Digestive Disease Research Institute, Tehran University of Medical Sciences, Tehran, Iran
[2]Bam University of Medical Sciences, Bam, Iran
[3]Institute for Health Metrics and Evaluation, School of Medicine, University of Washington, Seattle, USA

[#]A full list of authors is provided at the end of this paper

Supplementary Table 1. Covariates Used in CODEm Models of Overall Maternal Mortality.

Level	Covariate	Direction
**Level 1**	Age-specific fertility rate	+
	Total fertility rate (log-transformed)	+
	Maternal education (years per capita)	–
	In-facility delivery (proportion)	–
	Skilled birth attendance (proportion)	–
	Neonatal mortality ratio (log-transformed)	+
	Age-specific HIV mortality in females 10-54 (log-transformed)	+
**Level 2**	Antenatal care 1-visit coverage (proportion)	-
	Antenatal care 4-visits coverage (proportion)	-
	Age-standardised wasting (weight-for-height) summary exposure value (SEV)	+
	Age-standardised stunting (height-for-age) SEV	+
	Healthcare Access and Quality Index	-
	Age- and sex-specific SEV for high body-mass index (BMI)	+
	Age- and sex-specific SEV for high blood pressure (SBP)	+
	Underweight women of reproductive age	+
**Level 3**	Socio-demographic Index	-
	Mortality shock (cumulative rate in last 10 years)	+
	LDI (log-transformed)	-
	Hospital beds (per 1,000 population)	–

Supplementary Table 2. Age-standardised DALY Rates Per 100 000 due to Maternal Disorders in 1990 and 2019 and the Percent Change in Rates during this Time Period.

	**Age standardized DALY rates (95% UI) 1990**	**Age standardized DALY rates (95% UI) 2019**	**Percent change from 1990 to 2019**
Iran	210.4 (184.6, 237.3)	36.9 (31.7, 43.3)	-82.5 (-84.9, -79.7)
Alborz	156.8 (79.5, 223.5)	29.6 (22.5, 38.4)	-81.1 (-87.5, -61.2)
Ardebil	234.5 (139.2, 325.9)	32.3 (25.3, 41.1)	-86.2 (-90.5, -77.4)
Bushehr	341.4 (195.4, 476.6)	43.2 (34, 53.4)	-87.4 (-91.5, -77.2)
Chahar Mahaal and Bakhtiari	154.1 (107.1, 208)	25.5 (18.8, 33.2)	-83.4 (-88.5, -75.5)
East Azarbayejan	222.2 (152, 309.3)	33.2 (25.6, 43)	-85 (-90.4, -77.4)
Fars	219.6 (127.8, 310.6)	36.7 (28.4, 47.4)	-83.3 (-89, -70.3)
Gilan	140.4 (85.2, 194.4)	21.1 (16.5, 26.9)	-85 (-89.9, -74.7)
Golestan	199.8 (139.3, 271.5)	39.2 (31.1, 49.3)	-80.4 (-86.5, -70.7)
Hamadan	208.1 (153.8, 277.9)	33.3 (26, 42.4)	-84 (-88.9, -76.5)
Hormozgan	378.8 (137.4, 553.3)	52.3 (40.3, 66)	-86.2 (-91.2, -61.8)
Ilam	130.8 (90.1, 178.2)	20.8 (16.1, 26.8)	-84.1 (-88.7, -76.3)
Isfahan	164.3 (83.8, 229.8)	27.9 (21.7, 35.4)	-83 (-88.7, -63.6)
Kerman	296.2 (192.5, 419.6)	48.7 (37.8, 61.6)	-83.5 (-89, -74.4)
Kermanshah	266.7 (180.3, 373.4)	40.5 (30.9, 51.5)	-84.8 (-90.1, -75.5)
Khorasan-e-Razavi	326.4 (168.3, 457.8)	46.1 (36.2, 58)	-85.9 (-90.6, -72.8)
Khuzestan	210.3 (144.5, 290.6)	43.3 (33.8, 55.4)	-79.4 (-85.9, -69.2)
Kohgiluyeh and Boyer-Ahmad	278.7 (192.8, 381.9)	51.7 (38.7, 66)	-81.5 (-87.6, -70.7)
Kurdistan	292.8 (207.2, 402)	35.1 (27.5, 43.9)	-88 (-92, -81.5)
Lorestan	194.6 (134, 273)	32.1 (24.7, 40.8)	-83.5 (-88.8, -75.6)
Markazi	155.4 (106.9, 213.8)	23.8 (18.2, 30.2)	-84.7 (-89.6, -76.4)
Mazandaran	113.1 (81.5, 158)	21.4 (16.5, 27.5)	-81.1 (-86.8, -72.9)
North Khorasan	278.3 (204.4, 388.2)	38.8 (30.2, 48.4)	-86 (-90.2, -80.2)
Qazvin	236.6 (159.5, 325.7)	35.9 (27.7, 45.4)	-84.8 (-89.7, -74.9)
Qom	177.5 (72.5, 247)	26.1 (20, 33.6)	-85.3 (-90.1, -63.5)
Semnan	279.4 (162, 397.3)	40.2 (30.7, 50.8)	-85.6 (-90.8, -72.1)
Sistan and Baluchistan	449.8 (161.7, 667.8)	132.5 (101.4, 169.1)	-70.5 (-81.7, -23.6)
South Khorasan	261.3 (193.9, 345.8)	44.7 (34.7, 56.5)	-82.9 (-87.8, -76.4)
Tehran	120.7 (85.4, 167.5)	20.7 (15.4, 27.7)	-82.8 (-88.7, -74.8)
West Azarbayejan	267.9 (194.4, 363.5)	41.5 (32.3, 52.1)	-84.5 (-89.4, -78.3)
Yazd	224.6 (157.8, 309.2)	41.5 (31.6, 53.1)	-81.5 (-87.1, -72.6)
Zanjan	149.8 (106.8, 200.4)	22.7 (16.9, 29.3)	-84.8 (-89.4, -77.8)

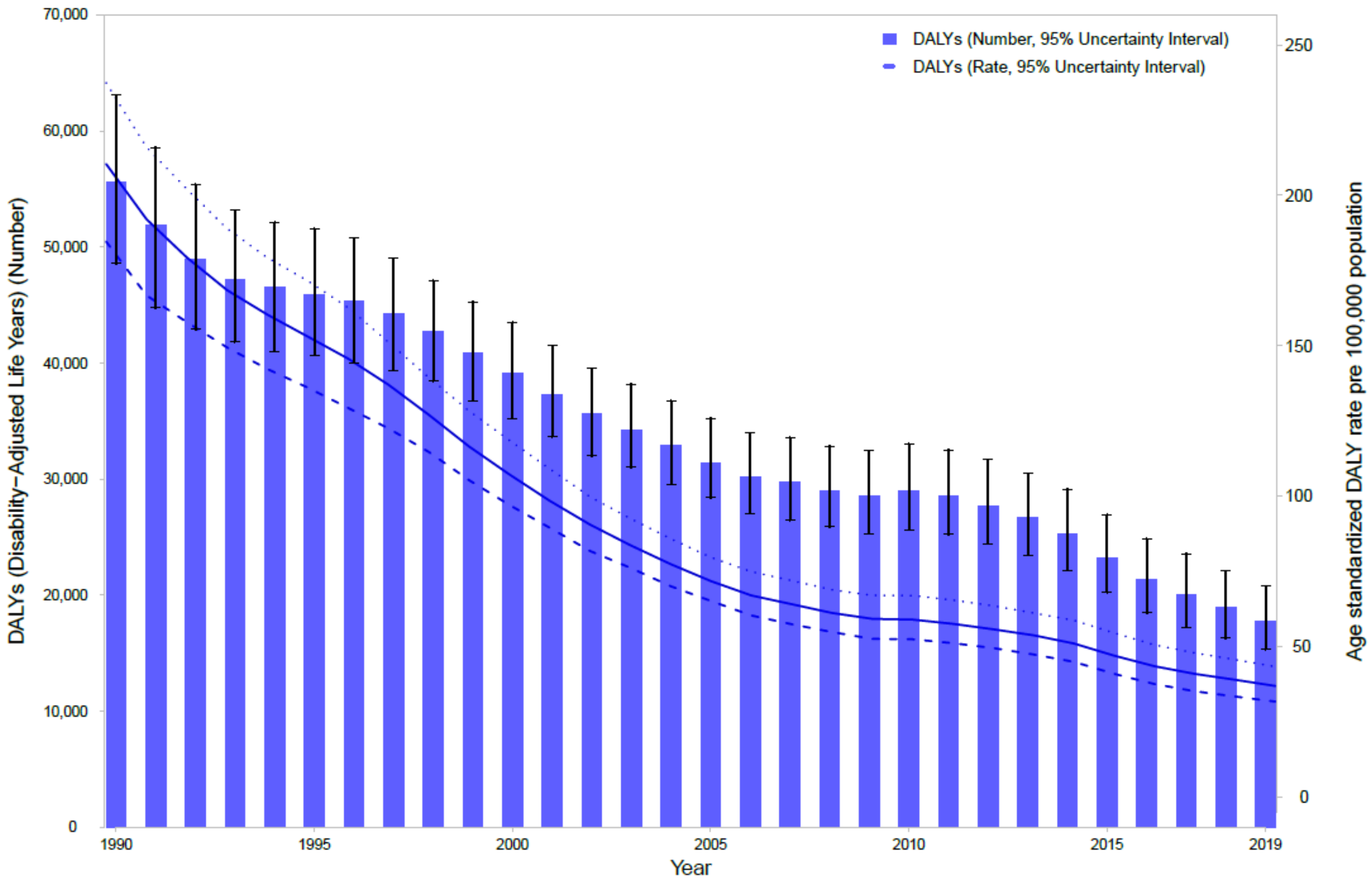

Supplementary Figure 1. The Trend in Number and Age-standardised DALY Rates per 100 000 due to Maternal Disorders from 1990 to 2019 in Iran.

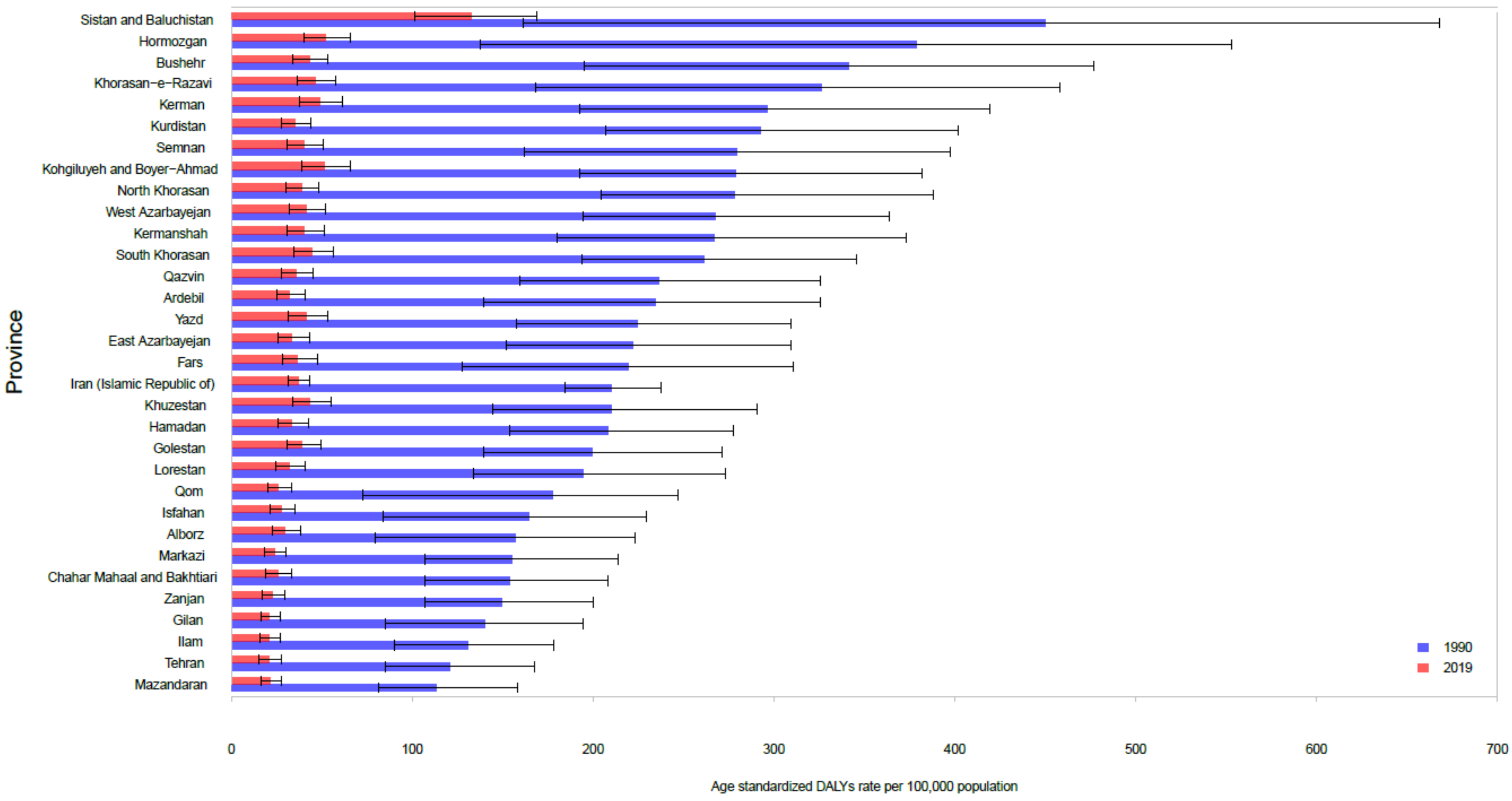

Supplementary Figure 2. Age-standardised DALY Rates due to Maternal Disorders per 100 000 across Provinces in Iran in 1990 and 2019.

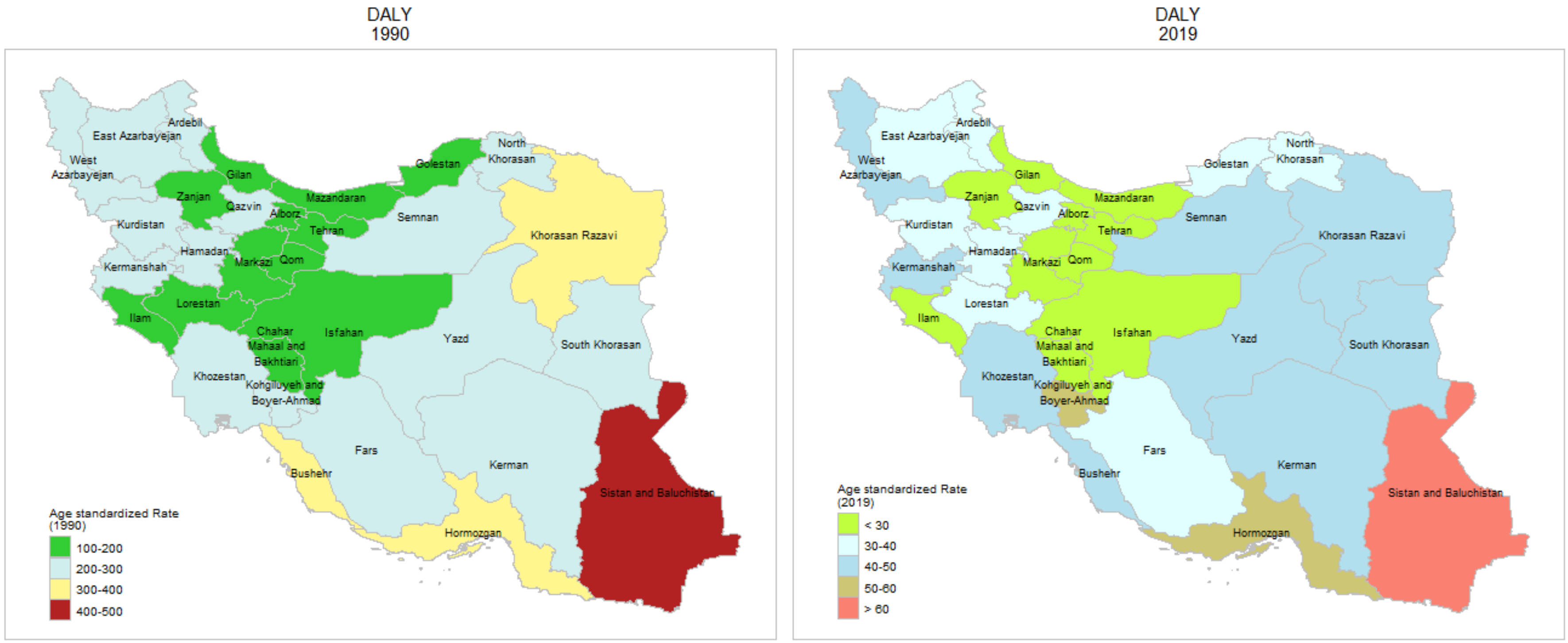

Supplementary Figure 3. Age-standardised DALY Rates per 100 000 due to Maternal Disorders across Provinces of Iran in 1990 and 2019.

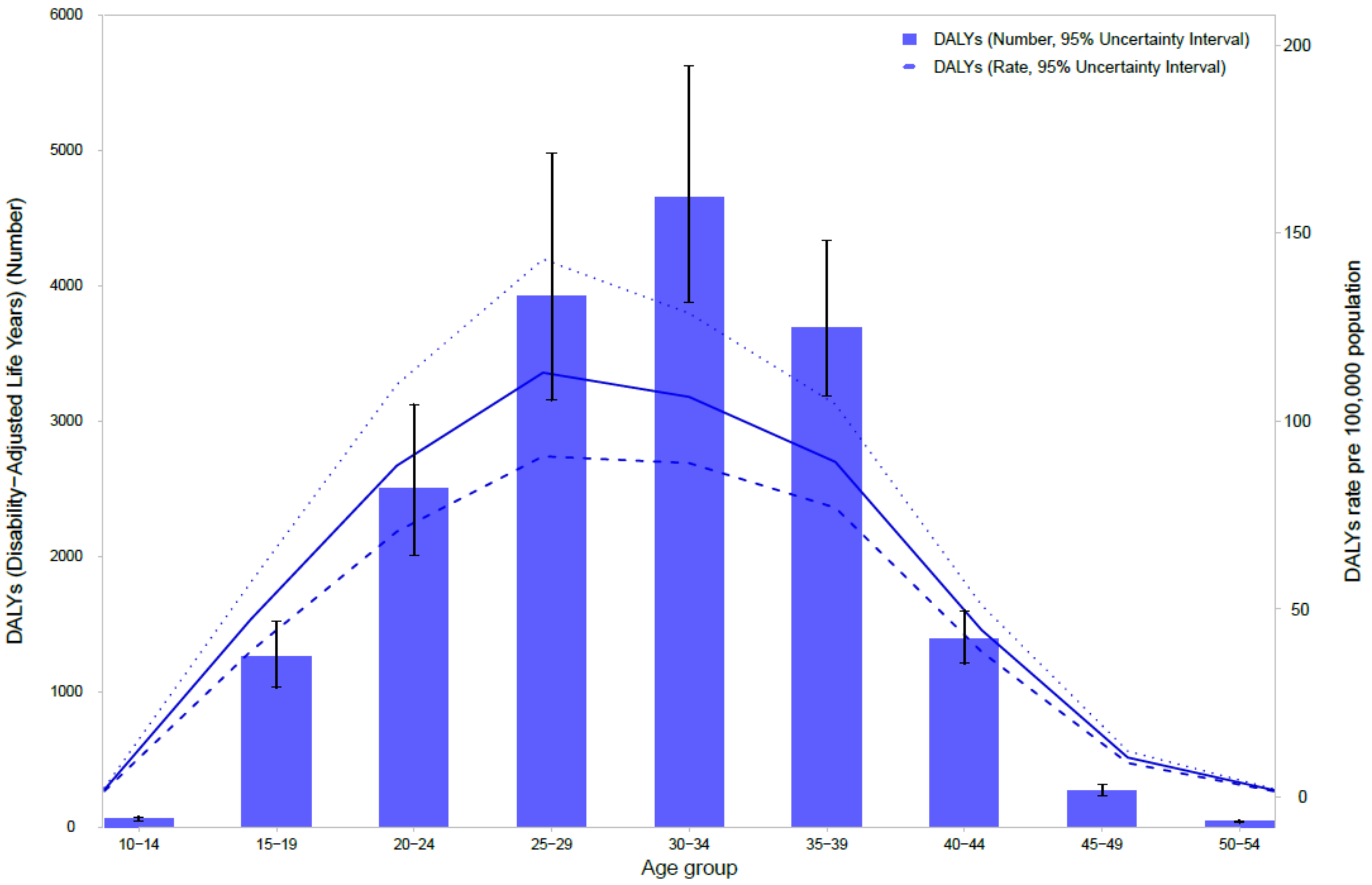

Supplementary Figure 4. The Age Pattern of DALY Numbers and Rates per 100 000 due to Maternal Disorders in 2019.

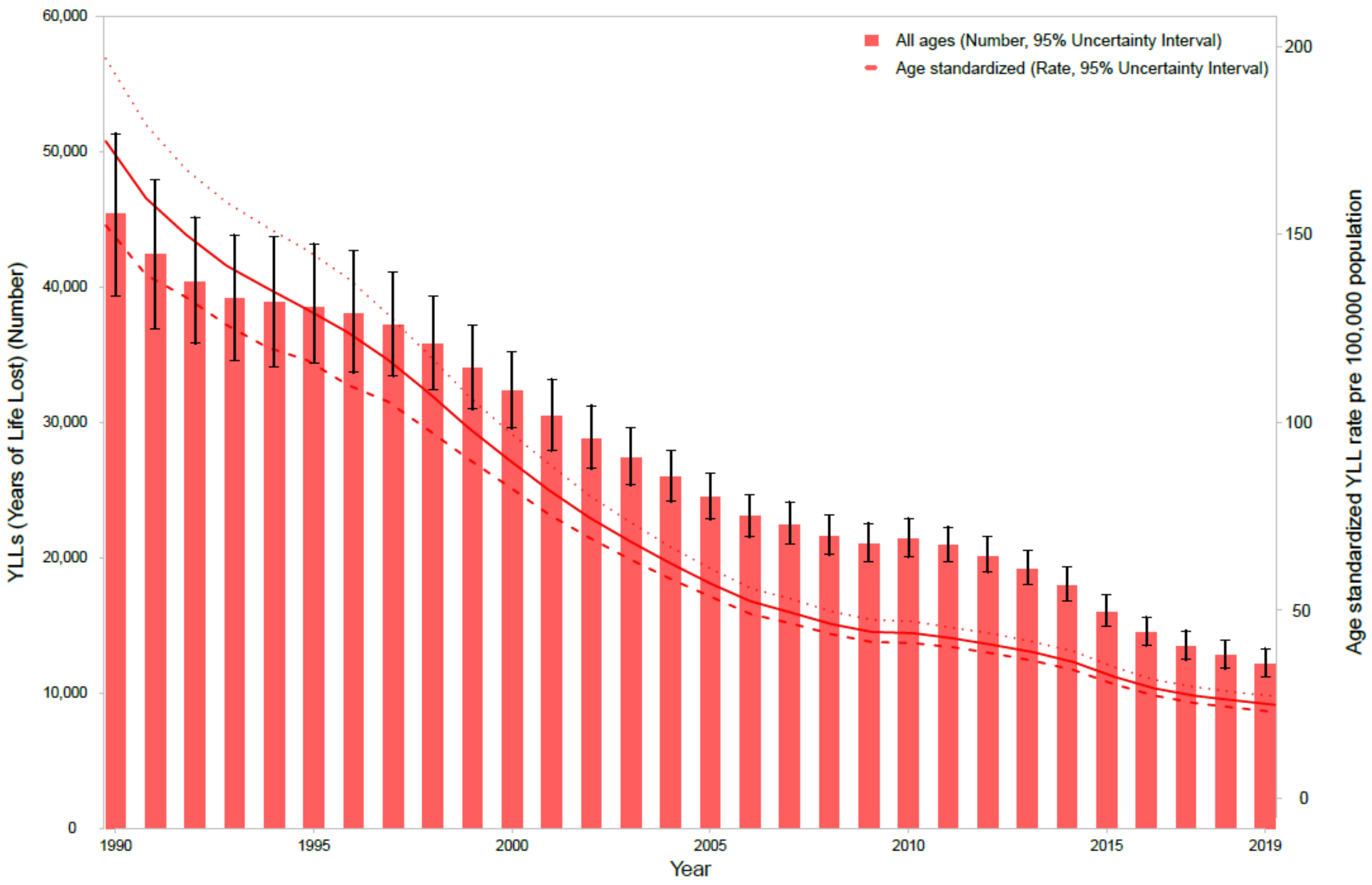

Supplementary Figure 5. The Trend in YLL Number and Age-standardised Rates per 100 000 due to Maternal Disorders from 1990 to 2019.

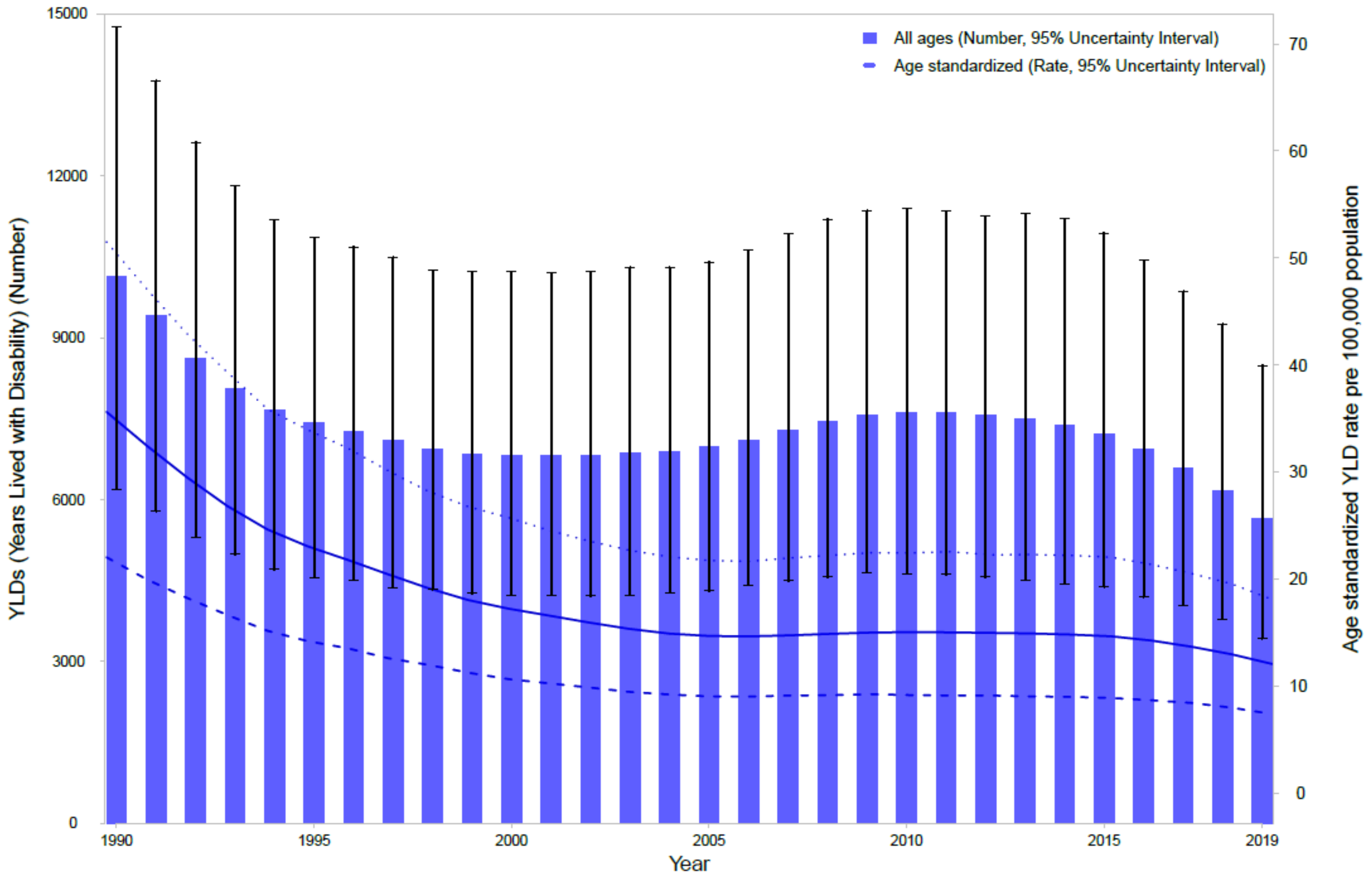

Supplementary Figure 6. The Trend in YLD Number and Age-standardised Rates per 100 000 due to Maternal Disorders from 1990 to 2019.

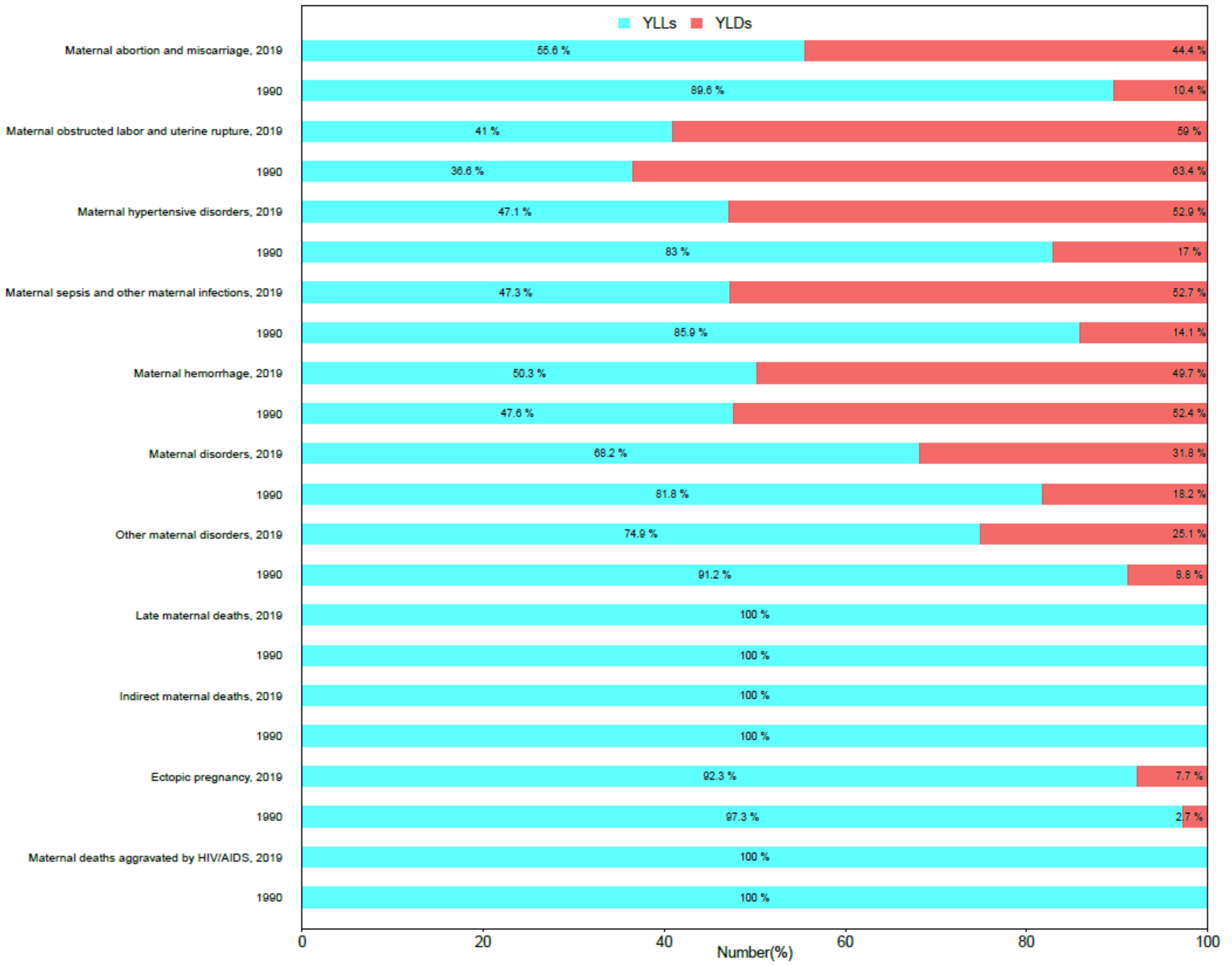

Supplementary Figure 7. The Share of YLLs and YLDs out of DALYs for Maternal Disorders by Cause in 1990 and 2019.

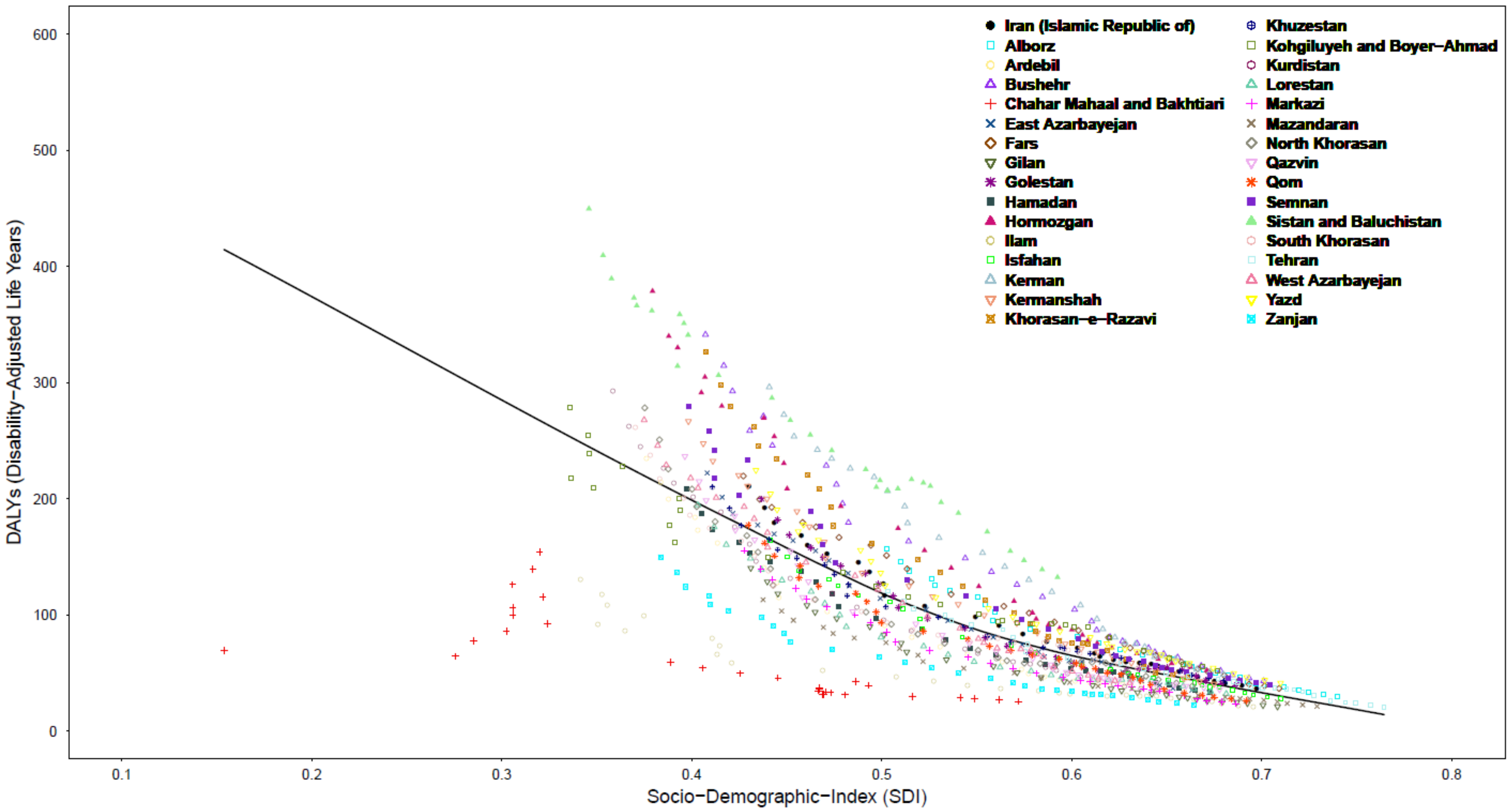

Supplementary Figure 8. The Trend in Age-standardised DALY Rates due to all Maternal Disorders across Provinces along with Increase in SDI from 1990 to 2019.
